# Editorial: Prognostic Research and Precision Oncology in Upper Tract Urothelial Carcinoma

**DOI:** 10.3389/fonc.2022.956352

**Published:** 2022-06-21

**Authors:** Zai-Lin Sheu, Nirmish Singla, Chung-Hsin Chen, Jer-Tsong Hsieh, Hsin-Chih Yeh

**Affiliations:** ^1^School of Medicine, College of Medicine, Kaohsiung Medical University, Kaohsiung, Taiwan; ^2^Brady Urological Institute, School of Medicine, Johns Hopkins Medicine, Baltimore, MD, United States; ^3^Department of Urology, National Taiwan University Hospital, Taipei, Taiwan; ^4^Department of Urology, University of Texas Southwestern Medical Center, Dallas, TX, United States; ^5^Department of Urology, Kaohsiung Municipal Ta-Tung Hospital, Kaohsiung, Taiwan; ^6^Department of Urology, Kaohsiung Medical University Hospital, Kaohsiung, Taiwan; ^7^Department of Urology, School of Medicine, College of Medicine, Kaohsiung Medical University, Kaohsiung, Taiwan; ^8^Graduate Institute of Medicine, College of Medicine, Kaohsiung Medical University, Kaohsiung, Taiwan

**Keywords:** upper tract urothelial carcinoma, precision oncology, symptomatic hydronephrosis, hematuria, pyruvate dehydrogenase kinase-3, acidic urine, pathology review, 7-(deoxyadenosin-N6-yl) aristolactam I

Upper tract urinary carcinoma (UTUC) is a rare but aggressive cancer, often leading to poor disease outcomes despite clinical interventions. While studies in UTUC have traditionally been limited by scarce case numbers and rapid disease progression, contemporary researchers have identified predictive and prognostic factors that could benefit treatment strategies. Several clinicopathological features of UTUC and their association with prognosis have been shown and endorsed by guidelines, including patient comorbidities, serum and urine markers, and pathological parameters such as tumor stage, tumor multifocality, and lymphovascular invasion (LVI) (1). Nevertheless, more precise biomarkers are still needed for earlier disease detection and wider utilization in clinical settings.

Eastern Asia in particular has a relatively high prevalence of UTUC, accounting for about 30% of all urothelial malignancies in this area, suggesting the presence of endemic risk factors on both environmental and genetic bases. In this Research Topic, five original articles based on East-Asian populations further explored UTUC prognosis from various angles, including its clinical manifestations, tumor biology, environmental factors, diagnostic methodology and risk factors, thereby providing deeper insights into precision oncology.

The most common and initial symptoms that UTUC patients exhibit are macro/micro hematuria and flank pain, the latter of which often arises from tumor obstruction in the urinary tract causing hydronephrosis. Previous studies have explored the impact of local symptoms, but the results were ambiguous. Within a large cohort of more than 2,500 cases, Yeh et al. successfully proved that these two local symptoms were inversely associated with disease outcomes. Symptomatic hydronephrosis and absence of hematuria both correlated with worse prognosis. This finding may benefit preoperative assessment and risk stratification for urologists to optimize disease management, since symptomatic hydronephrosis may prompt sooner or multimodal treatment strategies.

Deregulation of cellular energetics is a significant cancer hallmark. Within cancer cells, mitochondrial respiration is replaced by aerobic glycolysis for more rapid energy production. However, only few mitochondrial genes were investigated in urothelial carcinomas, including OXR1 (2) and ALDH2 (3). Kuo et al. found that high pyruvate dehydrogenase kinase-3 (PDK3) expression, detected by immunohistochemistry, is associated with adverse pathologic features and worse prognosis, including disease-specific survival and metastatic-free survival. This grants PDK3 the potential of predicting outcomes of UTUC. Besides participating in cellular metabolism, PDK3 might also have a role in tumor development, as evidenced by c0-upregulated genes that are associated with DNA replication and repair.

Aside from the tumor cells themselves, the tumor macro and microenvironments both influence their behavior. Acidic environments have been found to favor tumor motility, including invasion and metastasis. Hence, therapeutic manipulation of the tumor environment pH has been attempted in both hematological malignancies and solid tumors (4). Urothelial cancers are constantly exposed to urine, which is a potential acidic reservoir. However, urothelial studies regarding urine pH have been limited to bladder cancer patients. As a first for UTUC, a prospective cohort conducted by Han et al. found that acidic urine (defined as urine pH ≤ 5.5) is independently associated with poorer disease-free survival (DFS) and overall survival. This highlights the significance of the local environment in influencing prognosis in UTUC. In addition, this finding carries clinical value since urine pH test is a cheap, non-invasive and rapid method that could be easily measured.

Histopathologic interpretation of UTUC specimens plays a critical role in diagnosis and prognosis of UTUC, while potentially even informing treatment strategies for patients. However, interobserver variation may conceivably result in over staging or under staging of tumors. Chang et al. were the first to clarify interobserver variability in tumor staging and grading in UTUC. Fortunately, it had minimal impact on clinical practice. However, histopathological characters such as LVI and variant histology were subject to interobserver variations, underscoring the need for a more universal standard for future pathological review. A highlight of this study is the identification of intratubular spread of UTUC and its correlation with DFS. This adverse histologic feature was not uncommon in UTUC but was often neglected in studies. As a result, refinement of pathological review in UTUC might be a research direction in the future.

In another unique study, Lai et al. drew attention to the occurrence of UTUC in a population of kidney transplant recipients. Interestingly, end stage renal disease incidence, dialysis prevalence and UTUC occurrence after renal transplantation are all aberrantly high in Taiwan, suggesting a unique susceptibility of the upper urinary tract to nephrotoxicity and carcinogenesis in the Taiwanese population. A well-known toxin that is present in traditional Chinese medicine is aristolochic acid. Based on a unique genomic landscape, they found that faster UTUC onset after renal transplantation was predicted by a high level of 7-(deoxyadenosin-N6-yl) aristolactam I (dA-AL-I) detected in paired normal tissues. In addition, UTUC samples with dA-AL-I detection featured other genetic aberrations, including Signature 22 mutations, APOBEC-associated gene mutations, p53 mutations, mTOR activation, and high tumor mutational burden, unveiling the complicated mutational landscape of UTUC in kidney transplant recipients. Accordingly, for kidney transplant recipients exposed to aristolochic acid, dA-AL-I detection might be a useful and reliable biomarker to predict UTUC development after transplantation.

In this Research Topic, the authors shed new light on the prognostic landscape of UTUC. These findings hold important implications for clinical practice and may improve the management of patients with UTUC.

## Author Contributions

Z-LS drafted the editorial. NS and H-CY edited the manuscript. All authors contributed to this work and gave approval to the final version.

## Conflict of Interest

The authors declare that the research was conducted in the absence of any commercial or financial relationships that could be construed as a potential conflict of interest.

## Publisher’s Note

All claims expressed in this article are solely those of the authors and do not necessarily represent those of their affiliated organizations, or those of the publisher, the editors and the reviewers. Any product that may be evaluated in this article, or claim that may be made by its manufacturer, is not guaranteed or endorsed by the publisher.
